# A compact deep learning approach integrating depthwise convolutions and spatial attention for plant disease classification

**DOI:** 10.1186/s13007-025-01325-4

**Published:** 2025-04-02

**Authors:** Amreen Batool, Jisoo Kim, Yung-Cheol Byun

**Affiliations:** 1 Department of Electronic Engineering Institute of Information Science & Technology, Jeju National University, Jeju, 63243 Korea; 2 Faculty of Software, Artificial Intelligence Major, College of Engineering, Jeju National University, Jeju, 63243 Korea; 3 Department of Computer Engineering, Major of Electronic Engineering, Jeju National University, Jeju, 63243 Korea

**Keywords:** Agriculture, Plant leaf disease, Deptwise Spareabale convolutional, Spatial attention, MobileNet

## Abstract

Plant leaf diseases significantly threaten agricultural productivity and global food security, emphasizing the importance of early and accurate detection and effective crop health management. Current deep learning models, often used for plant disease classification, have limitations in capturing intricate features such as texture, shape, and color of plant leaves. Furthermore, many of these models are computationally expensive and less suitable for deployment in resource-constrained environments such as farms and rural areas. We propose a novel Lightweight Deep Learning model, Depthwise Separable Convolution with Spatial Attention (LWDSC-SA), designed to address limitations and enhance feature extraction while maintaining computational efficiency. By integrating spatial attention and depthwise separable convolution, the LWDSC-SA model improves the ability to detect and classify plant diseases. In our comprehensive evaluation using the PlantVillage dataset, which consists of 38 classes and 55,000 images from 14 plant species, the LWDSC-SA model achieved 98.7% accuracy. It presents a substantial improvement over MobileNet by 5.25%, MobileNetV2 by 4.50%, AlexNet by 7.40%, and VGGNet16 by 5.95%. Furthermore, to validate its robustness and generalizability, we employed K-fold cross-validation K=5, which demonstrated consistently high performance, with an average accuracy of 98.58%, precision of 98.30%, recall of 98.90%, and F1 score of 98.58%. These results highlight the superior performance of the proposed model, demonstrating its ability to outperform state-of-the-art models in terms of accuracy while remaining lightweight and efficient. This research offers a promising solution for real-world agricultural applications, enabling effective plant disease detection in resource-limited settings and contributing to more sustainable agricultural practices.

## Introduction

Agriculture is the backbone of human society, providing essential food, economic support, and raw materials for industries such as textiles and biofuels. Additionally, it plays a vital role in sustaining biodiversity and managing natural resources, contributing to economic stability and environmental sustainability. Plant diseases threaten agricultural productivity, leading to substantial financial losses and food security challenges worldwide. The early detection and management of plant diseases are essential to mitigate these impacts. Traditional methods for plant disease identification, such as expert visual inspection and laboratory-based techniques, have been widely used for decades. While effective in some instances, these conventional methods are often labor-intensive, time-consuming, and prone to human error. Furthermore, they may fail to detect diseases early, limiting interventions and increasing the risk of disease spread. These limitations underscore the need for more advanced, automated solutions to enhance the precision and efficiency of disease diagnosis in agricultural practices. It is a hot topic for researchers to detect and classify plant diseases to prevent crop loss and enhance food security. Convolutional neural networks (CNNs) have gained attention for their ability to detect and classify plant diseases from images of leaves accurately [1]. Spatial attention empowers the model to concentrate on pertinent regions within the input image, augmenting its ability to discern discriminative features [2]. The basic deep learning model Convolutional Neural Network (CNN) is used to classify plant leaf disease in detail in this review article [3].

Furthermore, CNNs are recognized for their efficacy in modeling intricate processes and conducting pattern recognition, particularly in applications dealing with substantial volumes of data, such as image pattern recognition [4]. A system based on Convolutional Neural Networks (CNNs) was introduced for the automated recognition of plants, explicitly using leaf images [5]. In [6], the author developed a robust neural network to successfully identify three distinct legume species by analyzing the morphological patterns of leaf veins. For the comparative analysis of two well-established CNN architectures in identifying 26 plant diseases [7]. Using an open database of leaf images from 14 different plants, the researchers achieved up to 80% success rates in automated identification [8]. However, the limitation was the exclusive use of images from experimental setups, which needed more representation of actual cultivation field conditions. To address a similar objective by developing a methodology for plant disease detection through leaf images, a comparable amount of internet-sourced data, with a smaller set of diseases in different plants, must be used. Success rates for their models ranged from 91% to 98%, contingent on the testing data. For the performance comparison between conventional pattern recognition techniques and CNN models for plant identification, using three diverse databases containing a limited number of images depicting entire plants and fruits or plant leaves [9].

Although some approaches have successfully recognized plant diseases, these processes require manual feature extraction, which can be complex and subjective. In this situation, it is difficult to identify the most reliable feature set. Traditional algorithms for recognizing plant leaf diseases have substantial hurdles due to the complicated concerns associated with plant diseases, which require detailed aspects of texture, shape, and color. This study proposes a lightweight deep learning architecture to address the challenges of multi-type detection of plant leaf diseases. Spatial Attention and Depthwise Separable Convolution are critical components of the proposed model. Depthwise Separable Convolution is a technique that improves model efficiency by separating spatial and depthwise convolutions, reducing the number of parameters and computation costs. On the other hand, Spatial Attention allows the model to focus on specific regions within the input image, enhancing its ability to capture essential and distinctive features of plant leaves. The combination of DWSC and SAM is designed to balance computational efficiency and diagnostic accuracy, addressing the constraints of real-world agricultural settings where resources are often limited. To ensure a robust and unbiased evaluation of the model, K-fold cross-validation is a reliable technique for assessing model performance. By partitioning the dataset into multiple folds and iteratively training and validating across these folds, the methodology ensures that all data points contribute to both training and validation. This minimizes the influence of random data splits, comprehensively evaluating the model capabilities. The novelty of this study lies between DWSC and Spatial Attention, which has not been widely explored for plant disease detection. While DWSC ensures computational efficiency, the spatial attention module refines feature extraction, allowing the model to achieve higher accuracy and robustness than traditional CNNs. This combination enables the LWDSC-SA model to outperform existing accuracy, precision, and recall methods while maintaining a lightweight architecture. The proposed method bridges the gap between high performance and practical applicability, making it a promising solution for automated plant disease detection in agriculture. The main contributions of this research are as follows:

- A novel lightweight, deep-learning model called LWDSC-SA has been developed to classify plant leaf diseases efficiently.
- The model incorporates multiple parameters and computational enhancements to improve overall classification efficiency, ensuring faster and more accurate disease detection.
- The LWDSC-SA model focuses on specific regions or patches of the leaf that are most relevant to disease classification, significantly reducing the computational cost. By concentrating on these targeted areas, the model enhances efficiency without compromising accuracy, leading to faster and more resource-efficient disease detection.
- The LWDSC-SA model was trained on the original plant leaf dataset, utilizing six standard data augmentation techniques for small datasets: random horizontal and vertical flips, central cropping, and adjustments in contrast, saturation, and brightness.
- The proposed LWDSC-SA model performance was evaluated using seven key metrics: accuracy, error rate, precision, recall, sensitivity, specificity, and F1-score. The results were benchmarked against six state-of-the-art deep learning image classification models: MobileNet, MobileNetV2, AlexNet, VGGNet-16, ResNet-50, and Inception V3, demonstrating superior performance in all categories.
- The model robustness and generalization capabilities were validated using K-fold cross-validation (K=5), providing a more thorough and reliable evaluation compared to traditional single data splits.

The article is structured as follows: Introduction section offers a brief introduction to leaf diseases, and Related work section reviews related work. The proposed model is outlined in Proposed methodology section , while Experiment setup and validation analysis section details the experimental setup and Results and discussion results and findings. The article concludes in Conclusion section.

## Related work

Plant disease classification is a crucial area of research due to its significant impact on agricultural productivity and food security. In recent years, integrating deep learning (DL) techniques has shown promising results in the accuracy and efficiency of plant disease detection systems. This section provides a comprehensive overview of relevant studies on plant disease classification, particularly those utilizing Depthwise Separable Convolution (DSC) and Spatial Attention mechanisms.

According to [10], the literature demonstrates the acceptability of many models in diagnosing various plant diseases. Using PlantVillage data created a unique architecture by integrating squeeze and excitation (SE) modules with deep learning [11]. In this architecture, the author uses pooling instead of a fully connected layer, improving the classification accuracy of 57.3 M parameters by 91.7%. In [12], the author found that the pre-trained CNN models B4 and B5, which were previously in use, outperformed additional models that were thought to be accurate CNN models. But B4 has 19 M parameters, and B5 has 30 M parameters. Therefore, more computation time is needed to increase the available resources. Several recent studies also use the attention mechanism to improve the effectiveness of various DL frameworks.

A study by [13] focused on the ResNet architecture for tomato plant disease diagnosis. They were able to detect ten tomato plant illnesses with 98% accuracy. In their Dense-Net CNN design, [14] integrated channel and spatial attention modules and substituted the Depthwise separable convolution operation. The datasets obtained from PlantVillage and their collection evaluated the usefulness of their method for diagnosing illnesses in maize plants. In the study, the author describes their gathered dataset, and the PlantVillage dataset model achieved 95.86% and 98.5% accuracy. created the RIC-NET model [15] utilizing the Inception and Residual blocks. Advancements in plant disease detection using machine learning (ML) and deep learning (DL), highlighting datasets, methodologies, challenges, and future directions [16]. A convolutional Block Attention Module (CBAM) was employed to improve the performance of the RIC-NET model. The authors’ model detected infections in tomato, corn, and potato plants with 99.55% accuracy. Author of [17] describes a novel architecture called Muti-Dilated-CBAM-DenseNet (MDCDenseNet) to detect maize plant diseases in farmlands. With maize plant leaf photos taken from Northeastern Agricultural University fields in China, their suggested model achieved 98.84% testing accuracy [18]. They also used the PlantVillage dataset to test the data and discovered that the SECNN model obtained 99.28% accuracy. Pandey and Jain [19] put forth a unique attention-based learning paradigm to enhance the CNN models to identify plant disease from leaf images. Ang et al. [20] introduced a Deep Hash Convolutional Neural Network (DHCNN) to enhance large-scale plant leaf disease retrieval. By leveraging collision-resistant hashing techniques and deep learning, the model achieved high precision 99.5% and F1-scores 99.58% on multi-plant disease datasets, demonstrating robustness and efficiency in retrieving highly similar plant disease images [21]. On the PlantVillage dataset, the accuracy of their suggested model was 99.93%. Using the DenseNet-121 CNN architecture operation, [22] achieved 98.17% accuracy in identifying plant diseases. Table 1 summarizes the findings from various studies, broken according to the number of classes, images, networks, and accuracy of the dataset given for the studies.

**Table 1:** A review of existing research and studies related to plant disease identification

Ref.	Dataset	Classes	Images	Aug	Network	Acc%
[23]	PlantVillage	14–36	54,309	Yes	VGG16	97.82
[24]	PlantVillage	06–10	54,306	Yes	Xception	97.35
[25]	Collected	15-6	4483	Yes	Modified CaffeNet	96.30
[26]	Collected	2-1	5808	Yes	Custom	95.83
[27]	Collected	9-1	1426	Yes	Two stage CNN	93.30
[28]	PlantVillage	20-01	9679	Yes	DLMC-Net	93.46
[29]	Collected	4-1	1053	Yes	Modified AlexNet	97.62
[30]	PlantVillage	3-1	3700	Yes	Modified LeNet	92.88
[31]	PlantVillage Collected	42-12	79,265	Yes	ResNet152	90.88
[32]	Collected	7-1	7905	Yes	Custom	90.16
[33]	Collected	9-1	5000	Yes	R-FCNN, ResNet50	85.98
[34]	PlantVillage	38-14	54,323	Yes	InceptionV3	95.30
[35]	PlantVillage Collected	56-14	2567	Yes	GoogleNet	94.00
[36]	Collected	6-1	6029	Yes	DenseNet+RF	79.59
[37]	Collected	10-1	500	No	Custom	95.48
[38]	PlantVillage Collected	10-1	17,929	N/A	F-CNN, S-CNN	97.60

Several studies have employed a variety of machine-learning algorithms and extensively classify plant diseases based on leaf images. Specifically, CAS-CNN and CAS-MODMOBNET models were applied in the cashew disease to evaluate the model performance with 99.8% accuracy [39]. Moreover, in [40], the author assessed modified transfer learning models (VGG19, NASNetMobile, DenseNet169) and state-of-the-art TL models (ResNet50V2, InceptionV3, Xception) for detecting potato leaf diseases. The modified DenseNet outperformed all models, achieving up to 99% accuracy and high MCC, CKC, and AUC-ROC scores. This study proposes the T-Net model, combining CNNs and transfer learning (VGG-16, Inception V3, AlexNet), achieving 98.97% accuracy for tomato disease detection, offering a rapid and reliable solution for farmers [41]. In a separate investigation involving tomato plants, a Support Vector Machine with a kernel-based function was applied to perform binary classification on 200 RGB images, achieving an impressive accuracy of 91.5% in distinguishing between two types of diseases [42]. Another robust model was developed for soybeans, capable of identifying, classifying, and quantifying a diverse range of foliar stresses. This model was trained on a substantial and varied dataset, with approximately 600 unseen test samples per class. The model demonstrated an overall classification accuracy of 94.13% [43]. However, these studies have several limitations, such as small and biased datasets and the diversity of the these datasets are not explicitly addressed. Furthermore, generalization of these models are still questionable as the synthetic samples fail to simulate the real-world scenarios, which leads towards over-fitting and poor generalization of these models and these models cannot be considered robust and resilient for unseen plant leaf disease detection scenarios. In addition, these traditional deep learning models are computationally expensive, potentially limiting their deployment on edge devices for real-time plant leaf disease detection. Therefore, a robust and lightweight approach is required to overcome aforementioned limitations for enhancing the plant leaf disease detection and classification. In this research study, a lightweight compact deep learning approach is proposed using depthwise separable convolutional and spatial attention techniques explicitly incorporated into MobileNet architecture initially introduced by Howard et al. in 2017 [44]. Our proposed approach uses depthwise separable convolutions consisting of depthwise and pointwise convolutions. In depthwise convolution, kernels from each filter convolve with individual input channels, whereas in pointwise convolutions, the resulting output channels are merged. This module generates a spatial attention map by considering the inter-spatial relationships present in the features. The spatial attention module helps the system in focusing on relevant spatial features, leading to a significant performance improvement while mitigating overfitting. Furthermore, our proposed approach architecture require less computational resources compared to the DenseNet, InceptionV3 and other tradtional DL architectures. In addition, our proposed study attempted to preprocess plant leaf images by incorporating photometric adjustments and geometric transformations in the augmentation process to mimic the variations and challenges that occur in real-world scenarios. In this way, we validate the proposed lightweight approach based on diverse dataset that simulate the real-world variations. The augmentation process simulates real-world variations to make the proposed approach more robust and resilient to efficiently cope with the unseen plant leaf disease detection scenarios. Moreover, we have compared the performance of our proposed approach with the state-of-the-art models to highlight the empirical effectiveness of our research study.

## Proposed methodology

**Fig. 1:**
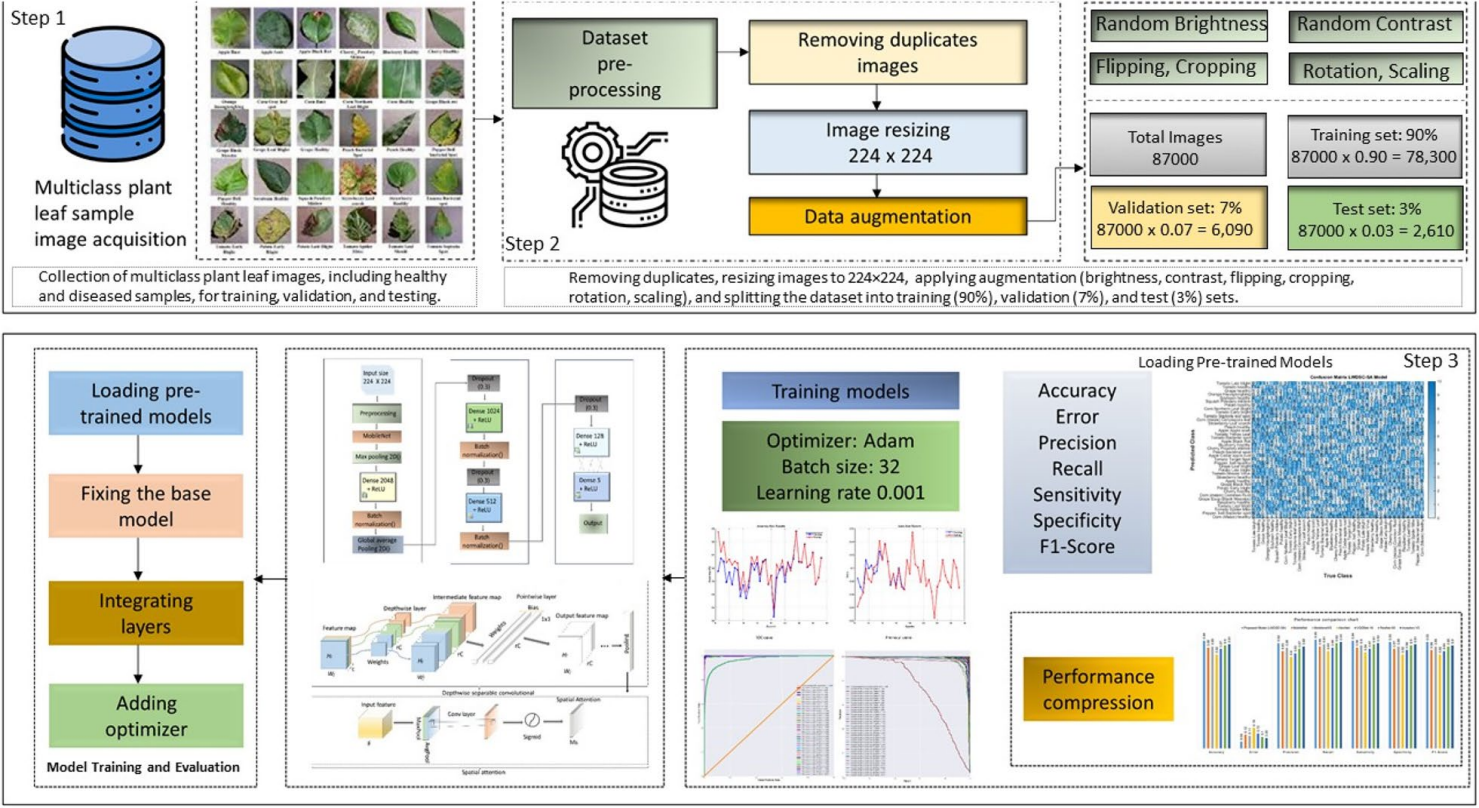
A comprehensive flowchart illustrating the systematic steps for identifying and diagnosing plant leaf diseases. The process includes image acquisition, dataset preprocessing (removing duplicates, resizing, and augmentation), training and validation using pre-trained models with optimized configurations, and final evaluation through performance metrics such as accuracy, precision, recall, and F1-score

This research proposes a framework for detecting plant leaf disease. The study aims to create an accurate and computationally less expensive learning model for disease diagnosis. This work proposes two different deep architectures for detecting disease in plant leaves. The first architecture integrates depthwise separable convolutional, and the second combines spatial attention architecture. Figure 1 represents a workflow for a plant disease identification system using machine learning. It starts with collecting plant images, then pre-processing and data augmentation to train a model. The system employs a Generative Adversarial Network and transfer learning for effective learning. After training and validation, the model classifies new images and updates the database, enhancing its accuracy over time.

### Image dataset

**Fig. 2:**
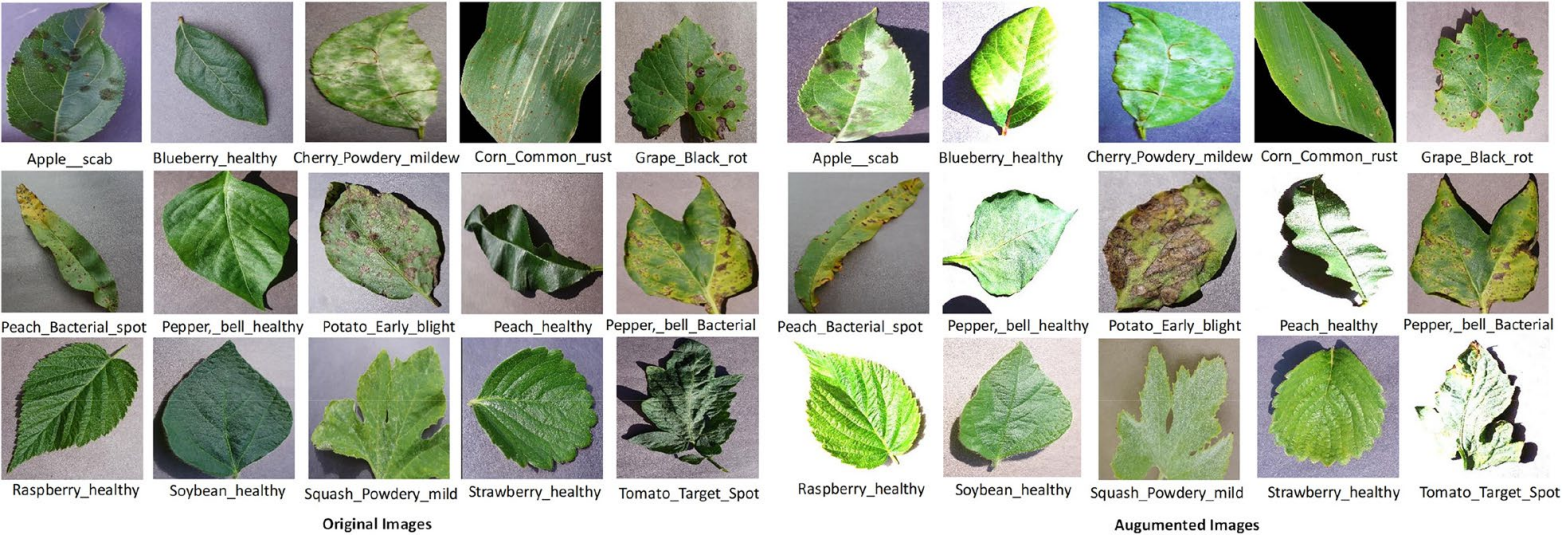
Sample original and augmented plant leaf images showcasing various disease categories and healthy leaves. The left section presents original images, while the right section displays corresponding augmented images generated through flipping, brightness adjustment, contrast adjustment, cropping, rotation, and scaling

The PlantVillage dataset, which features 55000 images and 38 classes, including 14 plant species, is used in this study. There are 26 classes corresponding to unhealthy plants and 12 classes corresponding to healthy plants; an additional class identifies 1,143 background images, resulting in 55000 images. Figure 2 displays a selection of 15 plant-disease pairs and healthy pairs randomly chosen. The author in [45] curated this dataset, featuring colored images of varying dimensions. To enhance the dataset, Geetharamani and Pandian [46] executed 6 augmentation methods and made both the original and augmented datasets publicly available; their augmentation strategy focused on increasing the sample size of classes with fewer than images through various augmentation techniques, while no modifications were made to images in other classes. Subsequently, the augmentation application resulted in a dataset expansion to 87000 images. The dataset is divided into training, validations, and test sets. For the study, the original and augmented datasets aim to enhance the performance of deep neural networks.

**Table 2:** Description of the plant leaf disease dataset, including class names, the number of images per class, and the distinction between healthy and diseased plant samples

Classes	Class name	Images	Classes	Class name	Images
0	Tomato_Late_blight	1851	19	Peach_Bacterial_spot	1838
1	Tomato_healthy	1926	20	Apple_rust	1760
2	Grape_healthy	1692	21	Tomato_Target_Spot	1827
3	Huanglongbing	2010	22	Pepper,_bell_healthy	1988
4	Soybean_healthy	2022	23	Grape_Leaf_blight	1722
5	Squash_Powdery	1736	24	Potato_Late_blight	1939
6	Potato_healthy	1824	25	Tomato_mosaic_virus	1790
7	Corn_Blight	1908	26	Strawberry_healthy	1824
8	Tomato_Early_blight	1920	27	Apple_healthy	2008
9	Tomato_leaf_spot	1745	28	Grape_Black_rot	1888
10	Corn_Cercospora	1642	29	Potato_Early_blight	1939
11	Strawberry_Leaf_scorch	1774	30	Cherry_healthy	1826
12	Peach_healthy	1728	31	Corn_rust	1907
13	Apple_scab	2016	32	Grape_Esca	1920
14	Tomato_Yellow_Leaf	1961	33	Raspberry_healthy	1781
15	Tomato_Bacterial_spot	1702	34	Tomato_Leaf_Mold	1882
16	Apple_Black_rot	1987	35	Tomato_Spider	1741
17	Blueberry_healthy	1816	36	Pepper_bell_spot	1913
18	Cherry_Powdery_mildew	1683	37	Corn_healthy	1859

### Data preprocessing

In this study, several key steps were involved in the data preprocessing. Initially, the input, the dataset, and the trim function control the dataset size and resize the images per class. This function regulated the sample count within 38 samples. Table 2 illustrates select images in the plant leaf disease dataset. Data augmentation is applied to extend the dataset by applying various photometric adjustments and geometric transformations to the original images. These photometric adjustments and transformations in augmentation process ensures to simulate a broad spectrum of possible real-world variations and enhance diversity in the synthetic dataset. Furthermore, we have considered environmental factors such as lighting which is simulated via random contrast in the augmentation process. In addition, these photometric adjustments and geometric transformations applied in the augmentation process to make our proposed model more resilient and adaptable to unseen scenarios, reducing overfitting and improving generalization to real-world data. The following photometric adjustments and geometric transformations are applied in the augmentation process such as:

- Random Contrast: Accounts for differences in sharpness and intensity.
- Horizontal and Vertical Flipping: Generates mirrored versions of images to introduce positional variations.
- Random Cropping: Extracts sub-regions to ensure the model recognizes disease features at different scales.
- Rotation and Scaling: Applies random rotations and scaling to emulate varying leaf orientations and sizes.

The dataset, including original and augmented images, was randomly split into three subsets. Training Set 90% This set is used for model learning and weight updates. Validation Set (7%): Employed for hyperparameter tuning and early stopping to prevent overfitting. Test Set (3%): Reserved exclusively for evaluating the final model performance on unseen data. Image Resizing and Normalization: All images are resized to the input dimensions of the respective models: 227 $\times$ 227 pixels for AlexNet, 224 $\times$ 224 pixels for ResNet50, VGG16, MobileNet, and LWDSC-SA. 299 $\times$ 299 pixels for Inception V3, Images were normalized by dividing pixel values by 255 to scale them within the range [0,1], ensuring compatibility with deep learning frameworks. For the evaluation model performance, K-fold cross-validation was employed. The dataset was divided into five equally sized folds, each serving as a validation set, while the remaining four were used for training. This iterative process was repeated five times, allowing every data point to be used for training and validation. K-fold cross-validation minimizes the impact of random splits and provides a more reliable assessment across the entire dataset. Table 2 provides an overview of selected sample images in the plant leaf disease dataset, showcasing healthy and diseased leaves across various categories.

### Model architecture

The proposed model is based on the depthwise separable convolutional neural network. It integrates spatial attention as a critical element into MobileNet, as explained by Howard et al. [44].

#### MobileNet

The study employs an improved version of MobileNet by adding extra layers to the existing MobileNet architecture [47]. Due to the limited available data and associated computational costs, transfer learning techniques help reduce the duration of training. MobileNet, a sturdy pre-trained model trained on the ImageNet dataset, comprises 28 convolution layers that function as the fundamental feature extraction component. One of its unique features is the use of depth-wise separable convolutions, which are nine times more computationally efficient than standard convolutions. MobileNet, known for its lightweight architecture and efficiency, has been widely used for image classification tasks, including plant disease detection. Recent studies, such as [39], demonstrated that MobileNet achieves high accuracy with minimal computational resources, making it suitable for real-world deployment in resource-limited environments.

**Fig. 3:**
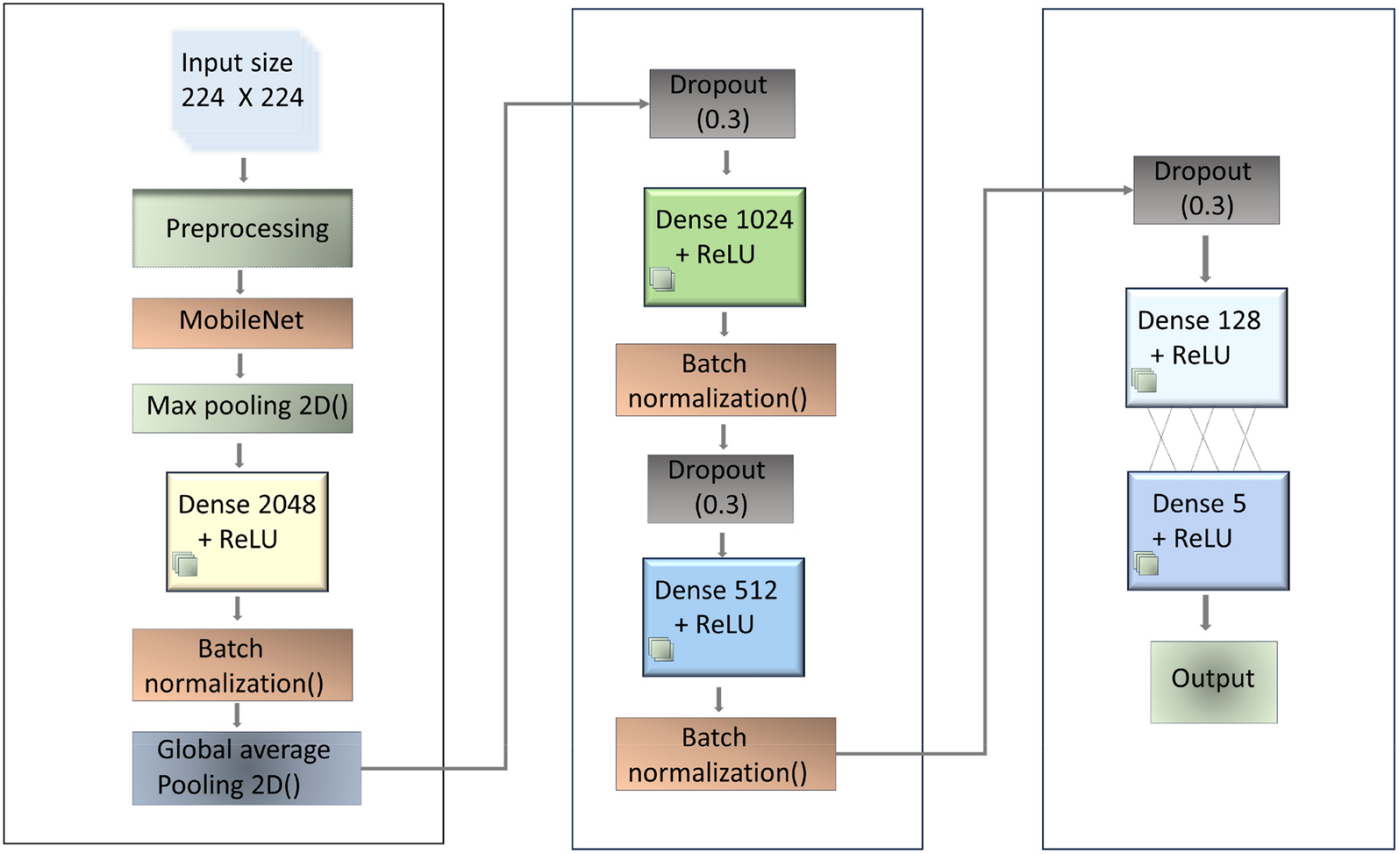
MobileNet-based model architecture with input preprocessing, depthwise separable convolutions, dense layers, dropout regularization, and output layer for plant disease classification

**Fig. 4:**
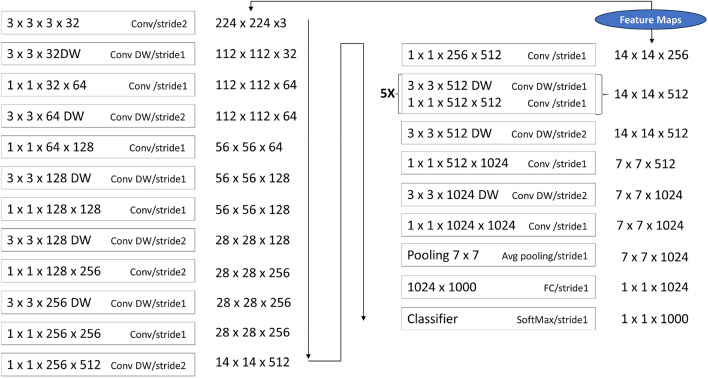
Detailed flow of depthwise separable convolutions, pointwise convolutions, and feature map generation. The architecture efficiently reduces computational complexity while maintaining performance, with feature maps transitioning from $224\times 224\times 3$ to $1\times 1\times 1000$, ultimately classified using the SoftMax layer

The MobileNet method involves factorizing a regular convolution into two components: a 3 $\times$ 3 Depthwise convolution and a 1 $\times$ 1 pointwise convolution. The model optimizes its size and speed by incorporating width and resolution multipliers. With this approach, a smaller and faster model can be developed, although it will lose moderate accuracy. Figures 3 and 4 depict the internal components of the architecture. It incorporates MobileNet to enhance the specific features of the data. Two max pooling layers and a global average layer are integrated, along with three batch normalization and dropout layers. ReLU serves as the activation function across four dense layers. The classification layer employs SoftMax, concluding with a final, lighter, dense layer. This composite design aims to optimize feature extraction, regularization, and classification within the framework while leveraging the efficiency of MobileNet.

#### Depthwise separable convolution

Depthwise Separable Convolution (DWSC) is an efficient form of convolution that factorizes a standard convolution into two sequential stages to reduce computational complexity and enhance performance. This two-step process comprises Depthwise Convolution (DWC) and Pointwise Convolution (PWC), as proposed by Sifre and Mallat [48].

*Depthwise Convolution (DWC)*: In this stage, a filter is applied independently to each channel of the input feature map to extract spatial features. It significantly reduces computation compared to conventional convolutions. Mathematically, DWC can be expressed as Eq. 1.

1 $$\begin{aligned} \operatorname {Depthwise \, Convolution}\left( W_d, y\right) _{(u, v)}=\sum _{h, l}^{H, L} W_{d(h, l)} \times y_{(u+h, v+l)} \end{aligned}$$

Here, *H* and *L* represent the height and width of the image, respectively. *u*, *v* represents the index position of the image, $W_d$ is the corresponding filter weights, and y denotes the input image.

*Pointwise Convolution (PWC)*: The output of DWC is then processed by a 1$\times$1 convolution across all channels. PWC combines the spatial features obtained from DWC, enabling interaction between channels The final outputs of PWC are considered the operation results of DWSC, as expressed in Eq. 2.

2 $$\begin{aligned} \operatorname {Pointhwise \, Convolution}\left( W_p, y\right) _{(u, v)}=\sum _m^M W_m \times y_{(u, v, m)} \end{aligned}$$

Where, $W_p$ filter weights for pointwise convolution and *M* Number of the input channels.

*Combined DWSC Operation* The final output is the combination of the two steps and is defined as Eq. 3:

3 $$\begin{aligned} \left( W_p, W_d, y\right) _{(u, v)} =\operatorname {Pointhwise \, Convolution}_{(u, v)}\left( W_p, \operatorname {DWC}_{(u, v)}\left( W_d, y\right) \right) \end{aligned}$$

DWSC reduces computational costs by separating spatial feature extraction (DWC) and channel-wise feature integration (PWC). This technique has been widely adopted in lightweight Convolutional Neural Networks (CNNs) such as MobileNets [44], Xception, ShuffleNet, and EfficientNets, where computational efficiency is critical.

**Fig. 5:**
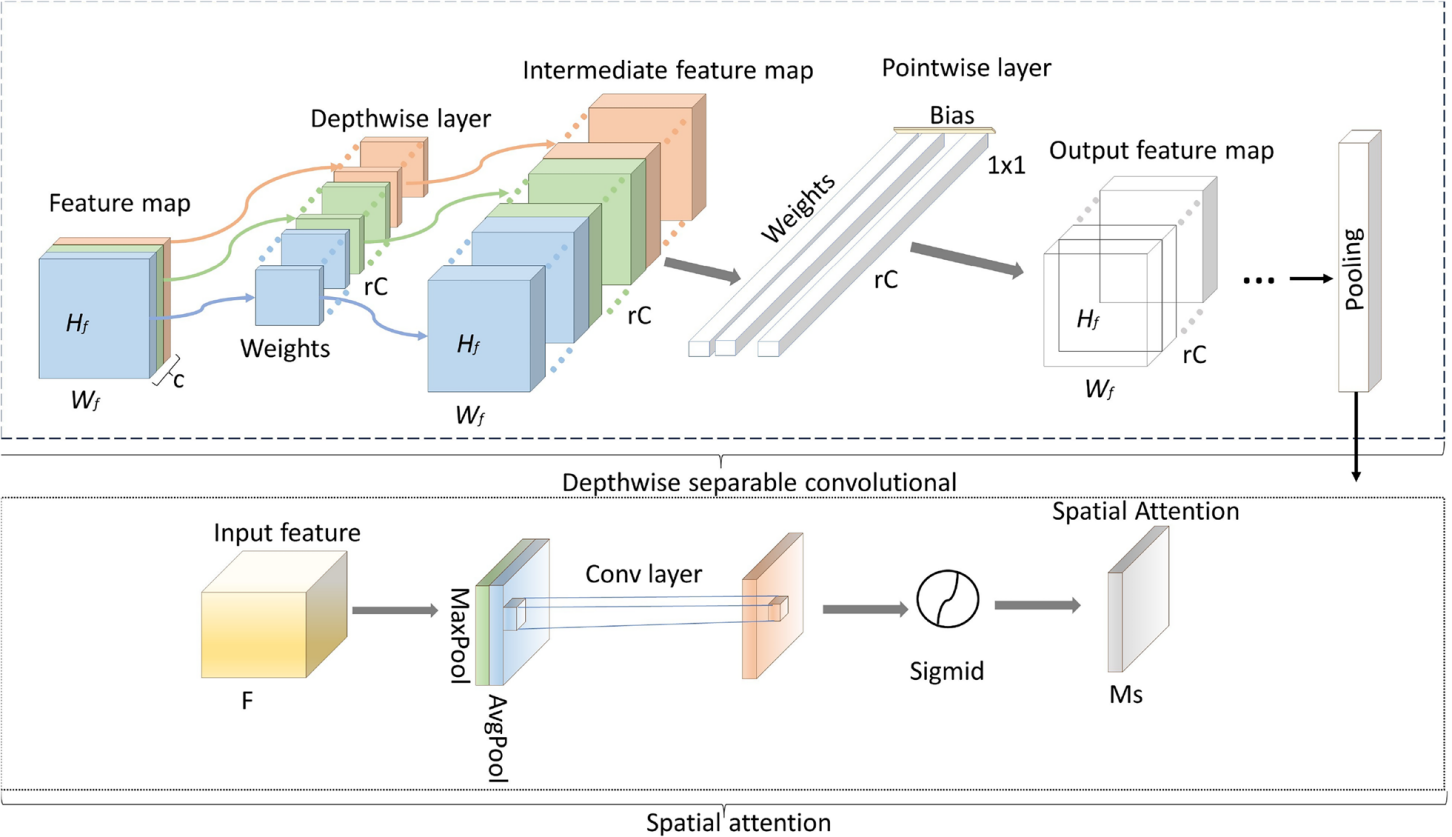
Illustrating the integration of Depthwise Separable Convolutional layers and Spatial Attention mechanism in neural networks, showcasing enhanced depthwise processing and spatial focus for improved feature extraction and model performance

#### Spatial attention

The Spatial Attention Mechanism refines the feature maps by highlighting spatially important regions while suppressing irrelevant background information. It enables the network to focus on disease-specific regions of plant leaves, improving accuracy in complex scenarios involving texture, shape, and color variations. *Input Representation*: Let *F* represent the input feature map: $F \in \mathbb {R}^{C \times W \times H}$ where *C*, *W*, and *H* are the dimensions of channel, width, and height.

*Spatial Attention Map*: SAM generates a 2D attention map $M_s$ by combining the outputs of Max Pooling and Average Pooling applied across the feature map *F*. SAM concatenates the final feature from channel attention and performs a convolution operation using a regular convolution layer, resulting in the spatial attention map computed by Eq. 4.

4 $$\begin{aligned} M_S(x)=\xi \left( f^{7 \times 7}([\operatorname {Maxpool}(F); \, \operatorname {Avgpool}(F)])\right) \end{aligned}$$

Where, $f^{7 \times 7} 7 \times 7$ convolution kernel applied to pooled features, $\xi$ Sigmoid activation function, * Element-wise multiplication.

*Improving Features *: The spatial attention map $M_s$ is multiplied with the original input *F* to produce the refined attention-weighted features. The comprehensive process of the attention module is expressed as Eq. 5.

5 $$\begin{aligned} F_{\text{ att } }=\operatorname {SAM}(F)=F * M_s(F) \end{aligned}$$

Here, the multiplication highlights the spatially relevant features, enhancing the model’s ability to detect disease regions in plant leaves.

SAM operates sequentially, where channel-wise and spatial-wise attention are computed consecutively. This sequential approach has been shown to achieve better performance in practical applications [49].

Figure 5 visually illustrates the integration of Depthwise Separable Convolution (DWSC) and Spatial Attention Mechanism (SAM) in the proposed architecture. The figure shows The separation of spatial feature extraction (DWC) and channel-wise feature integration (PWC) in DWSC. The attention map is generated in SAM through pooling and convolution operations, followed by the input feature map refinement. The combination of DWSC and SAM enhances the feature extraction capability of the model while maintaining computational efficiency. The proposed model achieves high performance with lower computational costs by focusing on disease-relevant regions and reducing redundant parameters. Depthwise Separable Convolutions reduce computational complexity, while Spatial Attention enhances feature interpretability, making the model ideal for low-resource agricultural environments. Furthermore,Table 3 presents a summary of the proposed model configuration parameters.

**Table 3:** Model configuration parameters

Type of layer	Parameters
Learning Rat	(0.001)
Optimizer	(AdaGard)
Batch Size	(32)
Dropout Rate	(0.5)
Image Size	(224 x 224)
Activation Function	(ReLU)
Loss Function	SC(cross-entropy)

Model configuration settings, including key parameters such as learning rate, optimizer, batch size, dropout rate, image size, activation function, and loss function. These parameters were optimized to enhance the performance of the LWDSC-SA model. Note: The loss function ’SC(cross-entropy)’ refers to Sparse Categorical Cross-Entropy, commonly used for multi-class classification tasks.

Furthermore, our proposed approach uses depthwise separable convolutions which involve fine-grained channel-wise operations, leading to sparse gradient updates. In the case of the sparse gradients, AdaGrad is efficient option compared to the Adam and SGD. Furthermore, AdaGrad ensures stable convergence without overshooting for our proposed approach having depthwise separable convolutions and spatial attention layers. Therefore, AdaGrad maintains a per parameter learning rate compared to the SGD, which relies on a fixed or manually decayed learning rate. In addition, Adam also uses adaptive learning rates by processing complex update rules such as momentum terms; therefore, it is not aligned with the aim of the depthwise separable convolutions to reduce the computational overhead as AdaGrad which avoid complex update rules. Therefore, AdaGrad is a well-justified choice for our proposed approach due to its compatibility with sparse gradients, lightweight design principles, and adaptive learning capabilities, particularly for architectures like MobileNet enhanced with depthwise separable convolutions spatial attention modules.

## Experiment setup and validation analysis

This section focuses on the experimental setup, model performances, and results, providing an in-depth analysis of our deep learning models on the dataset. The evaluation metrics considered include accuracy, precision, recall, and F1 score, offering a comprehensive understanding of the models per model. A comparison will be drawn between our pre-trained models and the LWDSC-SA model, and the outcomes of this research will be visually presented for a more precise illustration of the achieved results.

### Experimental setup

Table 4 provides detailed information about the hardware and software configuration used for running the experiments and training the model. The central processing unit (CPU) is an Intel Core i7-8700, operating at a base frequency of 3.20 GHz, offering strong multi-core performance for processing tasks. The graphical processing unit (GPU) is an NVIDIA GeForce GTX 1060 with 3GB of memory, ideal for handling computationally intensive tasks like deep learning. With 32 GB of RAM, the machine can handle big datasets and execute intricate processes without experiencing performance problems. A 500 GB hard drive serves as the storage, which is enough for managing data and installing software. It operates on a 64-bit operating system optimized for x64-based processors. The software version used is 22H2, which ensures compatibility with the latest tools and frameworks needed for model development. This setup provides an optimal balance of processing power and storage for efficient model training and experimentation.

**Table 4:** Tools and technologies used in the experiments conducted in this study

Configuration Item	Value
CPU	Intel(R) Core(TM) i7-8700 CPU @ 3.20GHz 3.19 GHz
GPU	NVIDIA GeForce GTX 1060 3GB
RAM	32.0 GB
Hard Disk	500 GB
Operating System	64-bit operating system, x64-based processor
Version	22H2

### Validation analysis

The confusion matrix is a widely used method for evaluating classification models. It provides insight into the number of correct and incorrect predictions by breaking them into True Positives (TP), True Negatives (TN), False Positives (FP), and False Negatives (FN). The model primarily aims to maximize TP and TN while minimizing FP and FN.

- *Accuracy* represents the overall effectiveness of the model, defined as the ratio of correct predictions to the total number of predictions made. It is calculated as follows:
  6 $$\begin{aligned} \text {Accuracy} = \frac{TP + TN}{TP + TN + FP + FN} \end{aligned}$$
- *precision* measures the proportion of correctly predicted positive observations out of all predicted positive observations. It is defined as:
  7 $$\begin{aligned} \text {Precision} = \frac{TP}{TP + FP} \end{aligned}$$
- *Recall*, also known as sensitivity, measures the proportion of actual positives correctly identified by the model. It is calculated as:
  8 $$\begin{aligned} \text {Recall} = \frac{TP}{TP + FN} \end{aligned}$$
- *F1 Score* is the harmonic mean of precision and recall, balancing the two metrics. It is beneficial when the class distribution is imbalanced. The F1 score is computed as follows:
  9 $$\begin{aligned} F1 = 2 \times \frac{\text {Precision} \times \text {Recall}}{\text {Precision} + \text {Recall}} \end{aligned}$$
- *Specificity* Specificity The proportion of correctly identified negative observations out of all negatives. It is expressed as:
  10 $$\begin{aligned} \text {Specificity} = \frac{TN}{TN + FP} \end{aligned}$$
- *Loss* expresses the difference between the expected and actual results. Standard loss functions for classification models include cross-entropy loss, which is used to optimize the model during training by reducing the difference between predicted and actual labels.

## Results and discussion

This section analyzes the proposed model performance through a series of experiments and evaluations. Starting with the Experimental Setup, we describe the technical environment and configurations used. The Training process is outlined, highlighting the optimization strategies and learning dynamics. Finally, the Results and Analysis provide a detailed discussion of the model performance, including key metrics and comparative evaluations against baseline models.

### Training

The datasets of PlantVillage, including the original and augmented versions. The augmentation techniques include random brightness, contrast, flipping, and cropping to enhance model generalization and robustness during training, divided randomly into training, validation, and test sets. The ratio used for the division was 90% for the training set, 7% for the validation set, and 3% for the test set. The training and validation datasets were only utilized for training and fine-tuning the model. At the same time, the test set was exclusively used to evaluate the model performance on samples that were unknown to the model. The study used existing DSC-SA models with transfer learning. The DSC-SA models were fine-tuned to identify and classify all categories in the dataset using pre-trained models on the ImageNet dataset to speed up the learning process. The last connected layers, which had multiple outputs, were changed to 38 to adapt the pre-trained models to the problem. All layers of pre-trained models were set as trainable. The activation function in the previous layer was set to Softmax, and the loss function was set to categorical cross-entropy. The early stopping technique was used during the training, with a patience value of 5 and a minimum change in loss of 1e-3. Since early stopping was used, the maximum epoch was not defined during model training. The models had already been trained using the same optimization method for training the ImageNet dataset. The VGG16 model uses the SGD optimization method, whereas all the other models use the Adam optimization method. The Adam optimization method was set to a learning rate of 0.001, while the SGD optimization method was set to 0.01. Adam optimizer with a learning rate of 0.001 was used for most models due to its adaptive learning capabilities, ensuring faster convergence. SGD optimizer with a learning rate 0.01 was used for the VGG16 model el, which benefits from SGD larger architectures.

**Fig. 6:**
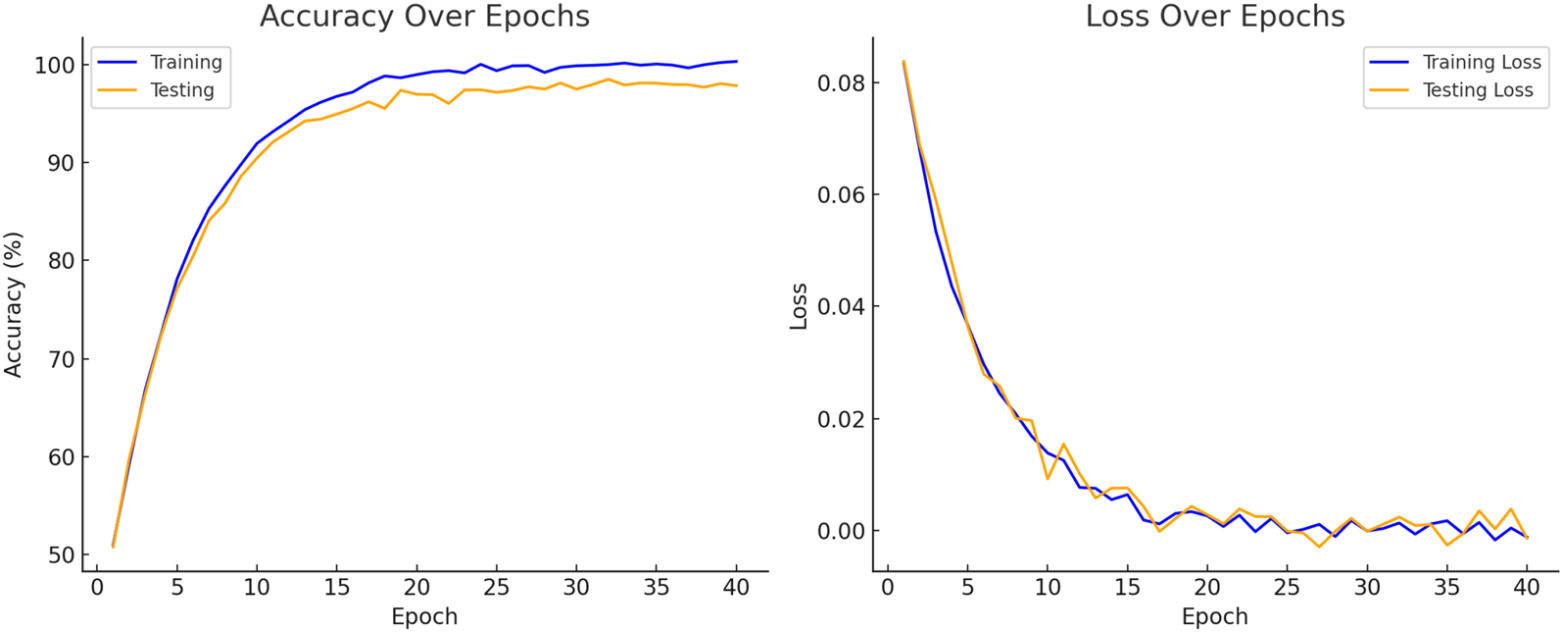
Training accuracy and loss accuracy of the model

The validation step for all models was set to 1. All images in the original and augmented datasets were first normalized by dividing by 255 and then resized to the default size accepted by each model. Accordingly, images were set to 227 $\times$ 227 pixels for AlexNet, 224 $\times$ 224 for ResNet50 and VGG16, and 299 $\times$ 299 pixels for Inception V3. Due to hardware limitations, input image resolutions were necessarily resized for all models of the MobileNet architecture in experimental studies. Figure 6 accuracy over epoch and loss of the model.

**Table 5:** Input image dimensions and total parameter counts for various deep learning models used in the study, highlighting their computational requirements and architectural differences

Model name	Input size	Number of total parameters
MobileNet	224 $\times$ 224	5,330,571
MobileNetV2	224 $\times$ 224	4,330,571
AlexNet	227 $\times$ 227	60,954,656
ResNet50	224 $\times$ 224	25,636,712
VGG16	224 $\times$ 224	138,357,544
Inception V3	299 $\times$ 299	3,851,784
LWDSC-SA	224 $\times$ 224	2,420,615

The maximum input size compatible with the hardware resources for the model with the highest parameter count was determined to be 224 $\times$ 224 pixels. To ensure uniform evaluation and fair comparison, the input size for all models, including MobileNet and its variants, was standardized to 224 $\times$ 224, except for AlexNet (227 $\times$ 227) and Inception V3 (299 $\times$ 299) due to their architectural requirements. Table 5 summarizes each image’s dimensions and corresponding total parameter counts. For the training process, a batch size 32 was used for all models, as this value balanced computational efficiency and resource utilization within the hardware constraints. The batch size was chosen to ensure consistent conditions across experiments while optimizing memory and processing capabilities. Table 6 details the key parameters used in the experiments, including image sizes, optimizers, and learning rates for each model. These standardized configurations ensured reliable and reproducible evaluations across all experiments.

**Table 6:** Configuration parameters for deep learning models, including input image size, optimization methods, and learning rates, highlighting the setup used for training and evaluating each model

Model name	Image size	Optimization method	L rate
MobileNet	224 $\times$ 224	$\text{ Adam } \text{ Optimizer } \text{(with } \beta _1=0.9 \text{ and } \beta _2=0.999 \text{) }$	0.001
MobileNetV2	224 $\times$ 224	$\text{ Adam } \text{ Optimizer } \text{(with } \beta _1=0.9 \text{ and } \beta _2=0.999 \text{) }$	0.001
AlexNet	227 $\times$ 227	$\text{ Adam } \text{ Optimizer } \text{(with } \beta _1=0.9 \text{ and } \beta _2=0.999 \text{) }$	0.001
VGG16	224 $\times$ 224	Stochastic Gradient Descent (SGD) with M = 0.0	0.01
ResNet50	224 $\times$ 224	$\text{ Adam } \text{ Optimizer } \text{(with } \beta _1=0.9 \text{ and } \beta _2=0.999 \text{) }$	0.001
Inception V3	229 $\times$ 229	$\text{ Adam } \text{ Optimizer } \text{(with } \beta _1=0.9 \text{ and } \beta _2=0.999 \text{) }$	0.001
LWDSC-SA	224 $\times$ 224	$\text{ Adam } \text{ Optimizer } \text{(with } \beta _1=0.9 \text{ and } \beta _2=0.999 \text{) }$	0.001

### Result and analysis

This section presents the results of the model evaluations; table 7 shows the average accuracy, recall, specificity, and values for each model based on the test datasets. The number of epochs selected for optimal performance for each performance criterion is divided by the total duration to determine the training time. The LWDSC-SA model, a novel approach to plant disease identification, introduces unique features that make it a promising solution for real-world agricultural settings with limited resources. Spatial attention and depthwise separable convolution of the model enhance the plant leaf images feature for accurate and efficient plant leaf disease and effectively address the challenges of complex agricultural conditions.

#### Confusion matrix of FWDSC-SA model

**Fig. 7:**
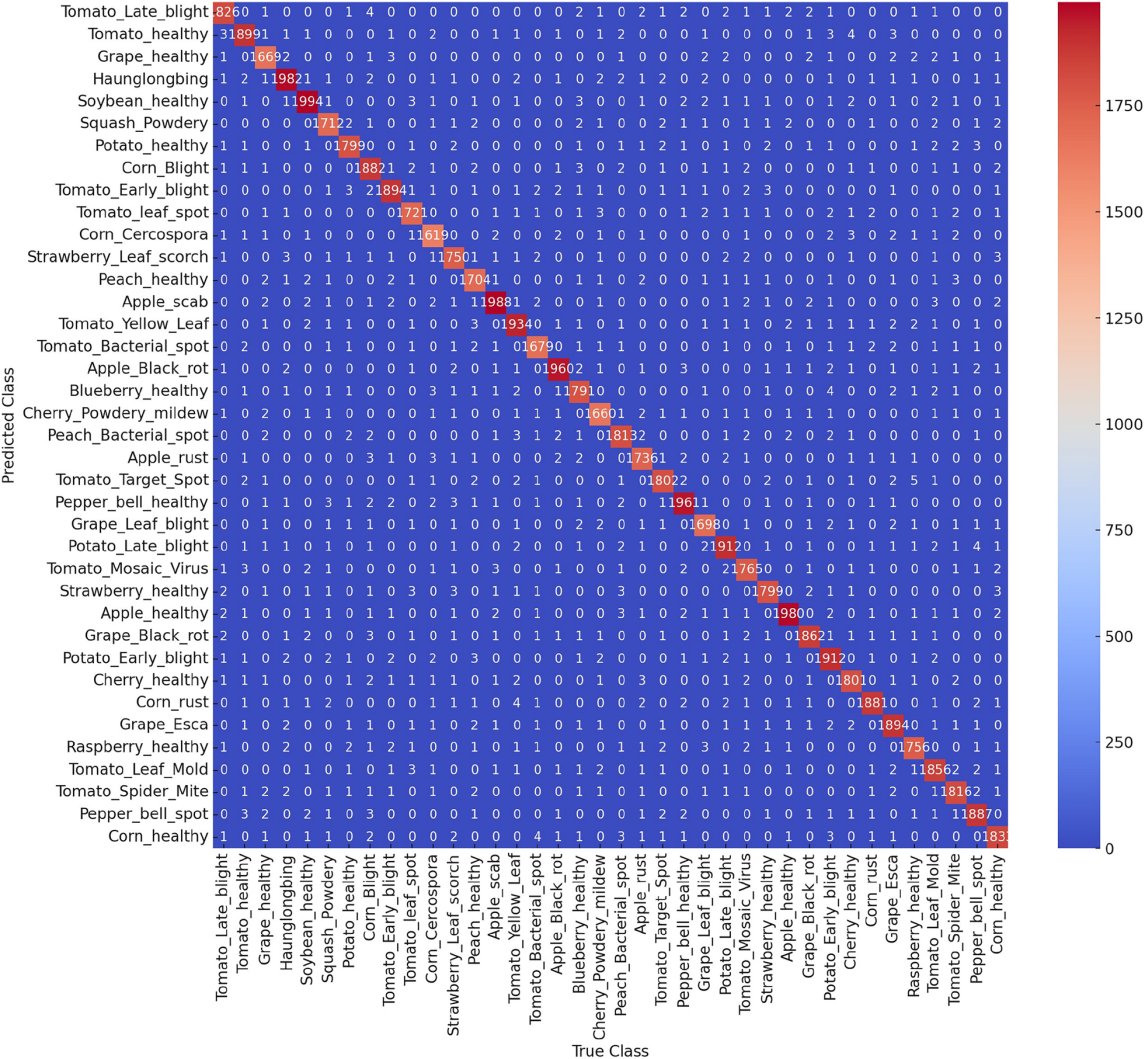
Confusion matrix of all plant leaf disease class

The proposed Lightweight Depthwise Separable Convolution with Spatial Attention (LWDSC-SA) model performance across several plant leaf disease classes is detailed in the confusion matrix presented in Fig. 7. The parallel values in the matrix indicate accurate classifications, and each row corresponds to the predicted class and each column to the actual class. The high values along the diagonal, particularly for classes like $Tomato\_Late\_blight$ (8326 correct predictions), $Tomato\_healthy$ (13891 correct predictions), and $Corn\_healthy$ (11887 correct predictions), indicate that the model performs exceptionally well in identifying these classes. These high values suggest the model is highly effective at learning discriminative features for diseased and healthy plant leaves, leading to high classification accuracy.

However, off-diagonal elements also represent misclassifications, though these values are relatively small compared to the diagonal values. For example, $Tomato\_Late\_blight$ was sometimes misclassified as $Tomato\_Leaf\_spot$ or $Tomato\_Early\_blight$, showing some confusion between different tomato disease types, which could be attributed to the visual similarities between these diseases. Despite these occasional misclassifications, the overall structure of the confusion matrix shows a strong performance, with most off-diagonal values being close to zero. The color scale, which ranges from blue to red, visually emphasizes this trend, as the diagonal elements are predominantly red, indicating high accuracy. This reflects the robustness of the LWDSC-SA model in distinguishing between multiple disease categories, even in complex cases where the visual differences between diseases may be subtle.

#### Performance analysis

The proposed Lightweight Depthwise Separable Convolution with Spatial Attention (LWDSC-SA) model showcases significant improvements over the conventional models used in plant disease classification, as demonstrated in Table 8. In terms of accuracy, the LWDSC-SA model achieved an accuracy of 98.70%, which is 5.25% higher than MobileNet (93.45%), 4.50% higher than MobileNetV2 (94.20%), 7.40% higher than AlexNet (91.30%), and 5.95% higher than VGGNet16 (92.75%). Compared to the other ideas, the improvement in accuracy shows how well the model can identify more complex patterns in the dataset, leading to a more accurate classification of plant diseases. Moving on to precision, the proposed model achieved an accuracy of 98.30%, representing a 5.50% increase compared to MobileNet (92.80%), 4.55% over MobileNetV2 (93.75%), 8.10% over AlexNet (90.20%), and a substantial 6.70% improvement over VGGNet16 (91.60%). This improvement in precision indicates that the LWDSC-SA model is better at reducing false positives, ensuring that it accurately identifies diseased plants while minimizing the incorrect classification of healthy plants as diseased.

The recall of the LWDSC-SA model also stands out, reaching 99.10%. This is a 5% increase over MobileNet’s recall of 94.10%, 3.50% over MobileNetV2 (95.60%), 6.65% over AlexNet (92.45%), and 5.25% over VGGNet16 (93.85%). This higher recall demonstrates that the LWDSC-SA model has an improved ability to detect diseased plants, minimizing false negatives and ensuring that nearly all instances of plant diseases are captured. The F1 score, which balances precision and recall, further emphasizes the model’s balanced and superior performance. The proposed model achieved an F1 score of 98.70%, which is 5.60% higher than MobileNet (93.10%), 4.20% higher than MobileNetV2 (94.50%), 7.40% higher than AlexNet (91.30%), and 6.10% higher than VGGNet16 (92.60%). This significant improvement reflects that the LWDSC-SA model effectively recognizes plant diseases and maintains a balance between sensitivity (recall) and precision.

**Table 7:** Performance analysis regarding accuracy, precision, recall, f1 score, specificity, and loss

Model	Accuracy	Precision	Recall	F1 score	Specificity	Loss
MobileNet	93.45	92.80	94.10	93.10	95.02	0.065
MobileNetV2	94.20	93.75	95.60	94.50	96.10	0.058
AlexNet	91.30	90.20	92.45	91.30	93.00	0.087
VGGNet16	92.75	91.60	93.85	92.60	94.20	0.072
Proposed model	**98.70**	**98.30**	**99.10**	**98.70**	**99.96**	**0.013**

**Accuracy**: Ratio of correctly classified instances to total instances. **Error**: Ratio of misclassified instances to total instances (1 - Accuracy). **Precision**: Proportion of accurate positive predictions out of all optimistic predictions. **Recall**: Proportion of correctly identified positives out of all actual positives. **Specificity**: Proportion of correctly identified negatives out of all negatives. **F1-Score**: The harmonic mean of Precision and Recall, balancing false positives and false negatives

Specificity is another critical metric for assessing how well the model can correctly identify healthy plants. The LWDSC-SA model achieved a specificity of 99.96%, the highest of all models. In comparison, MobileNet achieved 95.02%, MobileNetV2 96.10%, AlexNet 93.00%, and VGGNet16 94.20%. This results in an improvement of 4.94%, 3.86%, 6.96%, and 5.76%, respectively. This means the LWDSC-SA model is far less likely to incorrectly classify healthy plants as diseased. This is particularly valuable in practical applications where misclassification could lead to unnecessary treatments or interventions.

Lastly, the proposed model demonstrated the lowest loss value at 0.013, indicating more efficient convergence during training. Compared to MobileNet (0.065), MobileNetV2 (0.058), AlexNet (0.087), and VGGNet16 (0.072), the proposed model reduced the loss by 0.052, 0.045, 0.074, and 0.059, respectively. This drastic reduction in loss indicates a more stable and optimized training process, contributing to the model accuracy and generalization of unseen data. LWDSC-SA model demonstrates clear improvements across all key performance metrics. The enhancements in accuracy, precision, recall, F1 score, and specificity indicate that this proposed model is a more reliable and efficient tool for plant disease classification than traditional models, offering both accuracy and robustness for real-world agricultural applications.

**Fig. 8:**
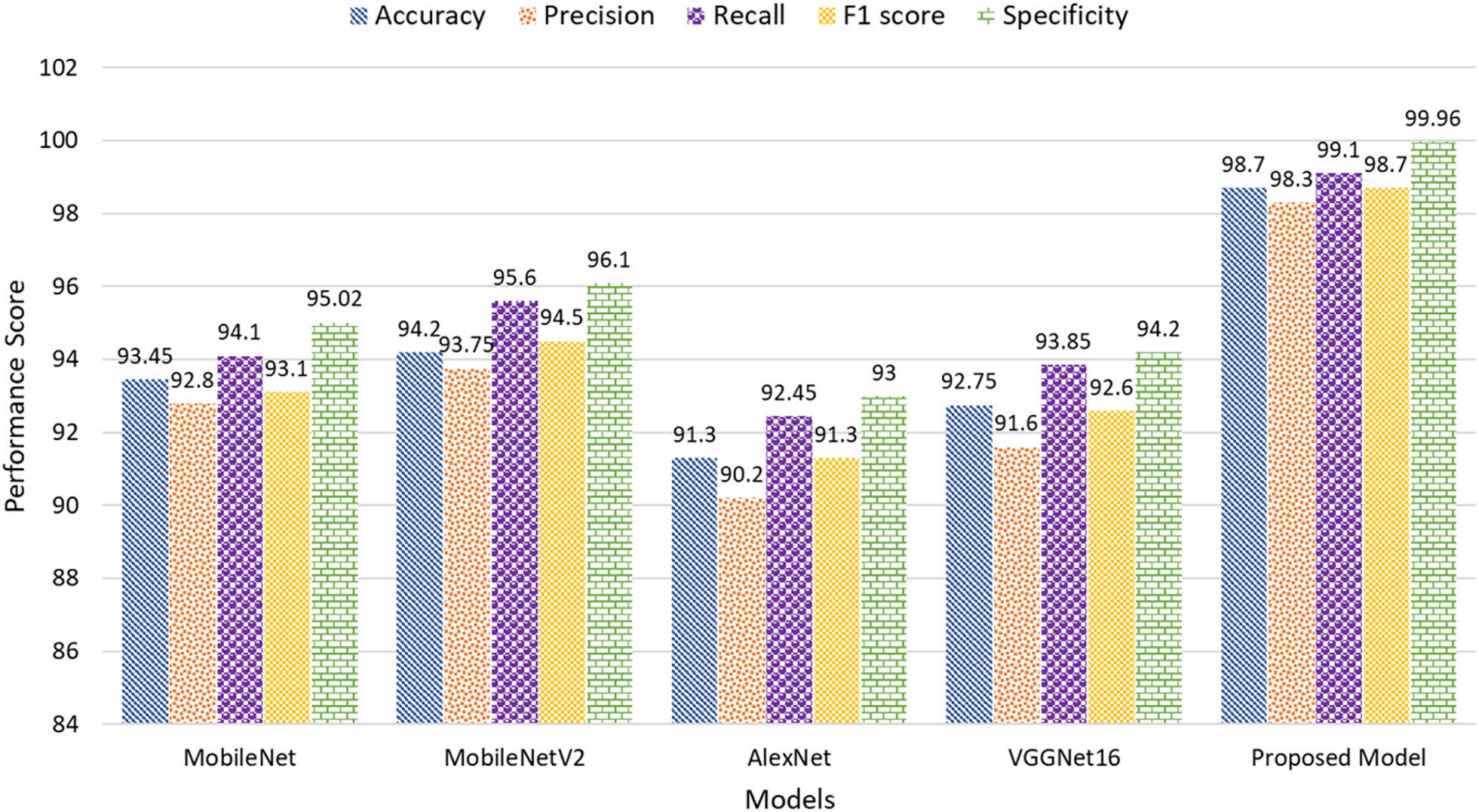
Performance analysis of the proposed model with the state-of-the-art models

Furthermore, the bar chart in Fig. 8 presents a comparative performance analysis of the proposed model against state-of-the-art models, including MobileNet, MobileNetV2, AlexNet, and VGGNet16. The proposed Lightweight Depthwise Separable Convolution with Spatial Attention (LWDSC-SA) model outperforms all other models across multiple metrics, including accuracy (98.7%), precision (98.3%), recall (99.1%), F1 score (98.7%), and specificity SpecificityCompared to MobileNetV2, the second-best performer, the proposed model achieves a higher recall and specificity its effectiveness in minimizing false positives and false negatives. These results demonstrate that the proposed model is more robust and efficient for plant disease classification, with improved performance across all key evaluation metrics.

**Fig. 9:**
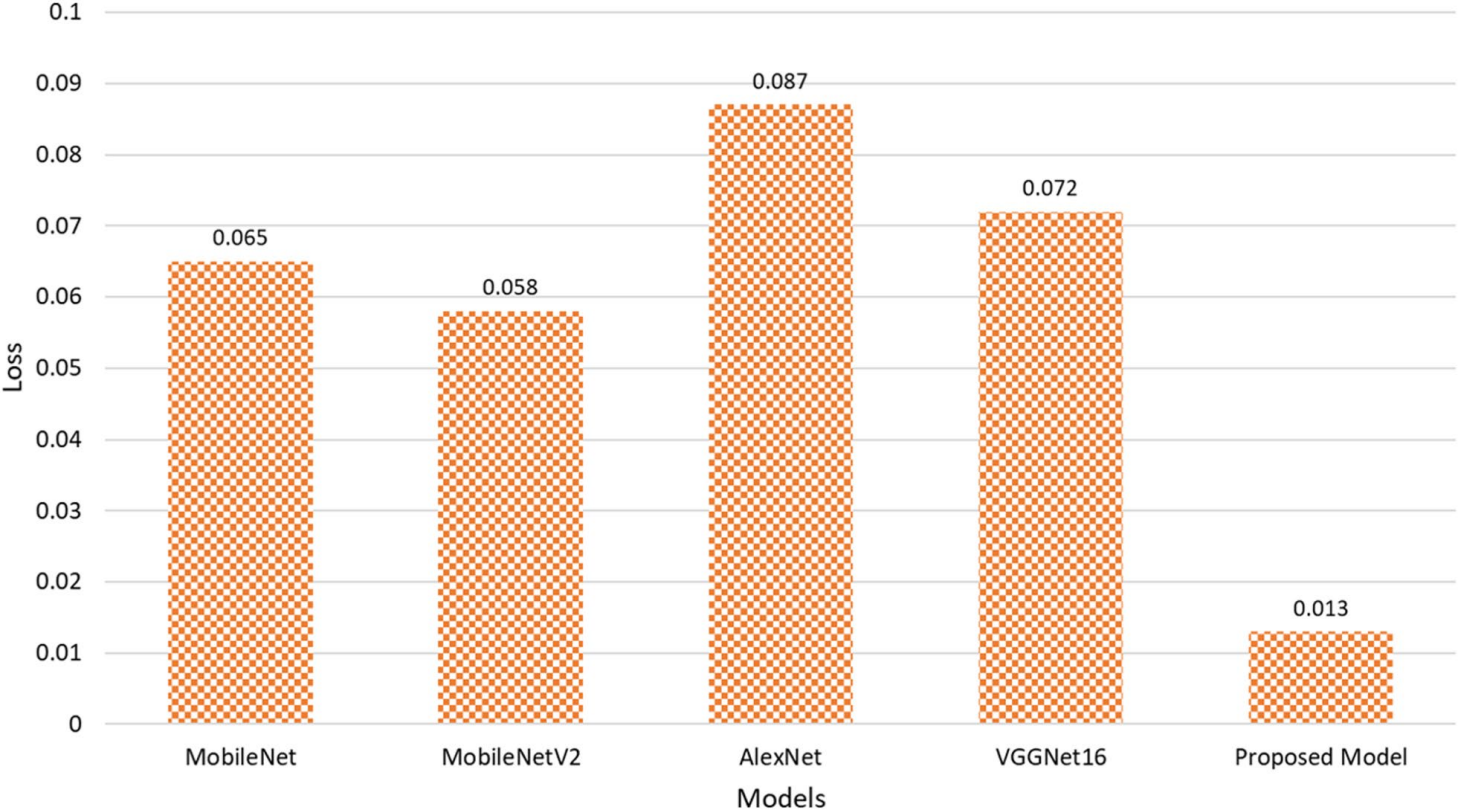
Performance analysis of the proposed model regarding loss value

Moreover, Fig. 9 illustrates the performance comparison of the proposed model against other state-of-the-art models in terms of loss value. The proposed Lightweight Depthwise Separable Convolution with Spatial Attention (LWDSC-SA) model achieves the lowest loss value of 0.013, significantly outperforming MobileNet (0.065), MobileNetV2 (0.058), AlexNet (0.087), and VGGNet16 (0.072). A lower loss value indicates that the proposed model is more efficient and stable during the training process, with a better ability to minimize prediction errors and generalize well to unseen data, ultimately leading to improved classification performance.

#### Statistical validation of LWDSC-SA model performance

To rigorously validate the performance improvements of the LWDSC-SA model over baseline models (MobileNet, MobileNetV2, AlexNet, and VGGNet16), paired statistical tests were conducted, as shown in Table 8. Mean differences in key performance metrics, including accuracy, precision, recall, F1 score, and specificity, were computed, revealing consistent improvements across all comparisons. For example, the LWDSC-SA model outperformed AlexNet by an average of 6.07% and MobileNet by 4.37% in these metrics. A paired t-test confirmed the statistical significance of these differences, with all p-values below 0.05 (e.g., for MobileNet). Additionally, 95% confidence intervals were calculated to provide robust estimates of the performance gains; for instance, the LWDSC-SA model’s improvements over MobileNet were estimated within a range of 2.46% to 6.29%. These results quantitatively affirm the LWDSC-SA model’s superior performance, demonstrating its robustness and reliability for plant disease classification, especially in challenging agricultural scenarios.

**Table 8:** Statistical validation of LWDSC-SA model compared to baseline models

Model	Mean difference (%)	t-Statistic	p-value	95% Confidence interval (%)
MobileNet	4.37	40.13	$2.30 \times 10^{-6}$	[2.46, 6.29]
MobileNetV2	3.43	20.77	$3.17 \times 10^{-5}$	[1.90, 4.96]
AlexNet	6.07	29.83	$7.52 \times 10^{-6}$	[3.40, 8.75]
VGGNet16	4.95	25.25	$1.46 \times 10^{-5}$	[2.76, 7.14]

#### Robustness evaluation using K-fold cross-validation

To further assess the robustness of the proposed LWDSC-SA model, we performed K-fold cross-validation, a widely accepted method to evaluate model performance comprehensively. This approach divides the dataset into K=5 equally sized folds, ensuring that each image is used for training and validation. The model is iteratively trained and validated k times, with one fold as the validation set, while the remaining k1 folds are used for training. This methodology provides a more thorough evaluation compared to the traditional single training-validation-test split, ensuring that the model is tested on all parts of the dataset. Additionally, this strategy minimizes the impact of random partitioning, leading to a more reliable assessment of the model’s capabilities.

The results from the K-fold cross-validation as shown in Table 9 demonstrate the high and consistent performance of the LWDSC-SA model across all metrics. The accuracy values for all folds remain tightly clustered around 98.5% to 98.7%, with an average of 98.58%. This indicates that the model can effectively classify plant diseases regardless of the data partitioning. Similarly, precision, recall, and F1 scores are consistently high, averaging 98.30%, 98.90%, and 98.58%, respectively. These metrics demonstrate the model’s performance in identifying true positives and avoiding false positives. The specificity value, averaging 99.28%, further supports correctly identifying negative cases, reducing the likelihood of misclassification. The loss values for each fold remain very low, ranging from 0.013 to 0.016, with an average loss of 0.0142. This indicates the model and minimal overfitting during training. Collectively, these results validate the robustness and generalization capabilities of the LWDSC-SA model, making it suitable for real-world agricultural applications.

**Table 9:** Performance metrics of the LWDSC-SA model using K-fold cross-validation

Fold	Accuracy (%)	Pre- (%)	Recall (%)	F1 Score (%)	Spec-(%)	Loss
Fold 1	98.5	98.1	98.9	98.5	99.2	0.014
Fold 2	98.7	98.4	99.0	98.7	99.4	0.013
Fold 3	98.6	98.3	98.8	98.5	99.3	0.015
Fold 4	98.4	98.2	98.7	98.4	99.1	0.016
Fold 5	98.7	98.5	99.1	98.8	99.4	0.013
Average	98.58	98.30	98.90	98.58	99.28	0.0142

### Discussion

The accurate and efficient classification of plant leaf diseases is essential for improving leaf disease and, minimizing the loss in agricultural production. Conventional models such as MobileNet, MobileNetV2, AlexNet, and VGGNet16 have shown varying levels of success in performing this task. Still, the increasing demand for higher precision, recall, and overall model accuracy requires more advanced approaches. The results from the graph above highlight the limitations of these models in terms of overall performance, demonstrating the necessity for a more efficient model that can deliver higher classification accuracy, particularly for real-time applications where robustness is key. The proposed Lightweight Depthwise Separable Convolution with Spatial Attention (LWDSC-SA) model addresses this challenge by introducing depthwise convolutions coupled with spatial attention mechanisms, optimizing feature extraction and model efficiency.

**Fig. 10:**
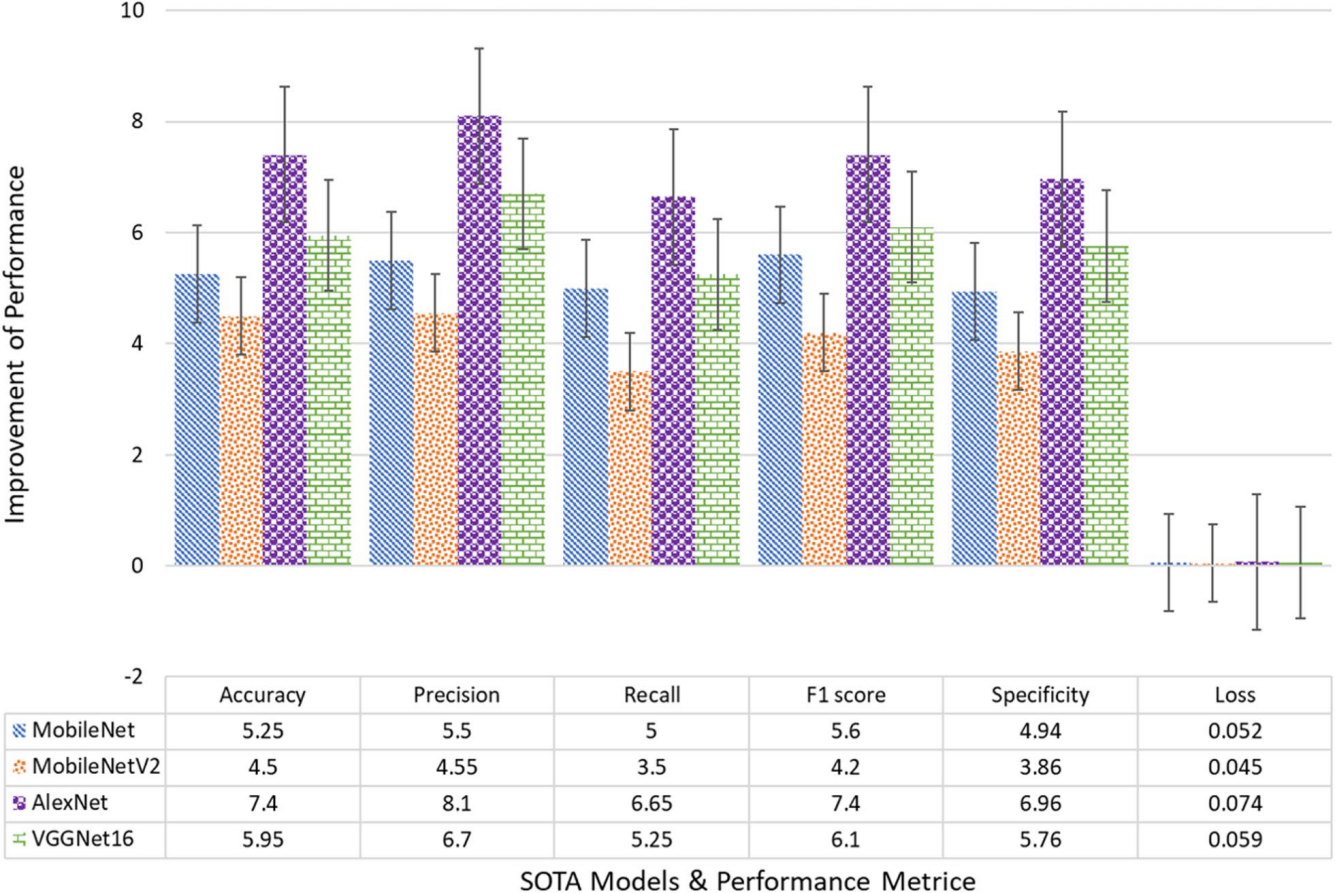
Performance analysis of the proposed model regarding Loss value

As shown in Fig. 10, the proposed model demonstrates substantial improvements over existing state-of-the-art models across multiple key performance metrics. For instance, compared to AlexNet, the proposed model improves accuracy by 7.4%, precision by 8.1%, recall by 6.65%, and F1 score by 7.4%, clearly showcasing its ability to outperform legacy architectures significantly. These improvements are particularly critical for models that accurately identify diseased leaves and minimize false positives. The specificity improvement is also noteworthy, where the proposed model outperforms MobileNet by 4.94% and AlexNet by 6.96%. Although the LWDSC-SA model performs exceptionally well in most classes, a few misclassifications were observed, especially among visually similar diseases such as Tomato Late blight, Tomato Early blight, and Tomato Leaf spot. These misclassifications highlight a limitation in the model’s ability to distinguish subtle visual differences in these specific categories. To address this, future work could refine the spatial attention mechanism to focus on disease-specific features. Additionally, including more diverse and class-specific data for these diseases could enhance the model’s ability to differentiate between them. Attention map visualization or feature attribution methods could also be employed to better understand the areas where the model struggles, leading to targeted improvements in its architecture.

Furthermore, the loss value improvements across all models underscore the efficiency and stability of the LWDSC-SA model during training. The proposed model achieves a drastically reduced loss of 0.013, compared to 0.074 for AlexNet and 0.065 for MobileNet, highlighting its ability to converge more effectively. This reduced loss results in more accurate predictions and suggests that the model generalizes well across different datasets and is less likely to overfit. Overall, the results affirm the significance of the LWDSC-SA model as a powerful tool for plant leaf disease classification, providing superior performance across a range of crucial metrics while maintaining computational efficiency.

A key strength of the proposed model lies in its lightweight design. With only 2.4 million parameters, the LWDSC-SA model significantly reduces the computational burden compared to VGG16 (138 million) and ResNet50 (25 million). This efficiency makes the LWDSC-SA model ideal for deployment on low-power devices such as mobile phones, edge devices, and drones. In real-world agricultural settings, where access to high-performance hardware is often limited, this lightweight architecture enables real-time disease detection, empowering small-scale farmers to take timely action and minimize crop losses.

The LWDSC-SA is attributed to its thoughtful combination of DWSC and SAM, optimizing feature extraction and computational performance. Its ability to focus on critical regions in plant images while reducing redundant computations sets it apart from existing models. The LWDSC-SA significantly improves accuracy, precision, and recall, while its lightweight design ensures practical applicability in real-world scenarios.

**Fig. 11:**
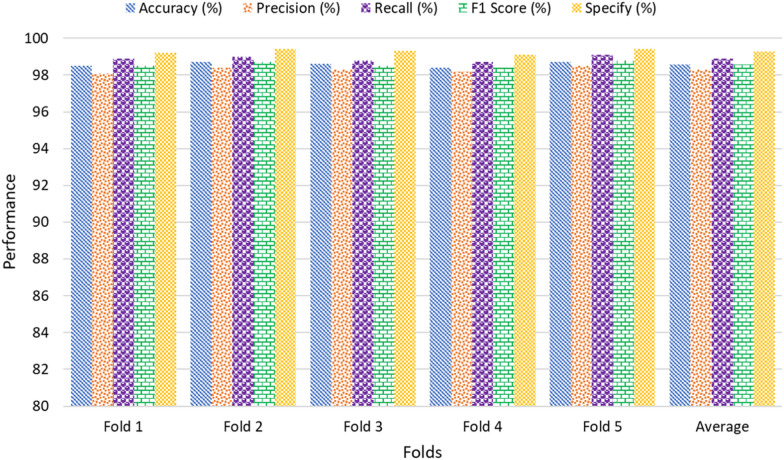
Performance analysis of the proposed model using 5-fold cross-validation

The bar graph in Fig. 11 visualizes the performance metrics of the LWDSC-SA model across the five folds and their average, showcasing high and consistent performance across all metrics. Accuracy, precision, recall, F1 score, and specificity grouped, exceeding 98% in all folds, with minor fluctuations between them, further emphasizing the model. Notably, specificity achieves the highest values across the folds, reaching an average of 99.28%, reflecting the model correctly identifying negative cases. The minimal variance observed in the metrics across folds highlights the robustness and generalization capabilities of the LWDSC-SA model, confirming its effectiveness in handling different subsets of data and reinforcing its potential for real-world applications.

## Conclusion

In conclusion, accurately detecting plant leaf diseases is critical for enhancing agricultural productivity and mitigating crop losses. While effective, traditional deep learning models like MobileNet, MobileNetV2, AlexNet, and VGGNet16 face challenges extracting intricate features from plant images and often lack the computational efficiency required for real-world applications. To address these limitations, we proposed the Lightweight Depthwise Separable Convolution with Spatial Attention (LWDSC-SA) model, which integrates depthwise separable convolutional layers and spatial attention mechanisms to capture fine-grained features such as texture, shape, and color while maintaining a lightweight design. The LWDSC-SA achieved significant improvements over state-of-the-art models, with an accuracy of 98.7%, precision of 98.3%, recall of 99.1%, F1 score of 98.7%, and specificity while demonstrating a reduced loss of 0.013. These results highlight the model’s robustness, efficiency, and suitability for deployment in resource-constrained environments, making it ideal for real-world agricultural applications. Despite its promising performance, the proposed work has certain limitations. First, the dataset used in this study, while extensive, may not fully represent real-world variations such as complex backgrounds, varying lighting conditions, and leaf occlusions. Addressing these factors would enhance the diverse agricultural environments. Future research can focus on expanding the dataset to include more plant species and environmental conditions and exploring various geographical regions and crop types, further enhancing its potential as a versatile tool for precision agriculture. The LWDSC-SA model presents a promising solution for improving early and accurate plant disease detection, ultimately contributing to global food security and agricultural sustainability.

## Data Availability

The dataset utilized in this study is the PlantVillage dataset, which is publicly available and widely used in plant disease detection research. For more details and to access the dataset, please visit the official repository at this link.
